# Enhancing human capillary tube network assembly and maturation through upregulated expression of pericyte-derived TIMP-3

**DOI:** 10.3389/fcell.2024.1465806

**Published:** 2024-10-31

**Authors:** Ksenia Yrigoin, Kaitlyn N. Bernard, Maria A. Castaño, Ondine Cleaver, Saulius Sumanas, George E. Davis

**Affiliations:** ^1^ Department of Molecular Pharmacology and Physiology, Morsani College of Medicine, University of South Florida, Tampa, FL, United States; ^2^ Department of Molecular Biology, UT Southwestern School of Medicine, Dallas, TX, United States; ^3^ Department of Pathology and Cell Biology, Morsani College of Medicine, University of South Florida, Tampa, FL, United States

**Keywords:** endothelial cells, capillaries, pericytes, vascular smooth muscle cells, basement membrane matrix deposition, mural cells

## Abstract

In this study, we identify and characterize new molecular determinants that optimize human capillary tube network assembly. Our lab has previously reported a novel, serum free-defined 3D co-culture model using human endothelial cells (ECs) and human pericytes whereby EC-lined tubes form and co-assemble with pericytes, but when these cultures are maintained at or beyond 5 days, tubes become progressively wider and unstable. To address this issue, we generated novel human pericytes that carry a tissue inhibitor of metalloproteinase (TIMP)-3 transgene which can be upregulated following doxycycline addition. EC-pericyte co-cultures established in the presence of doxycycline demonstrated marked enhancement of capillary network assembly including dramatic narrowing of capillary tube widths to an average of 8 µm (physiologic capillary tube width), increased tube lengths, increased tube branching, and robust stimulation of basement membrane matrix assembly, particularly with collagen type IV and fibronectin deposition compared to controls. These substantial changes depend not only on induction of pericyte TIMP-3, but also on recruitment of pericytes to EC tubes. Blockade of pericyte recruitment prevents these dramatic capillary network alterations suggesting that EC-pericyte interactions and induction of pericyte TIMP-3 are necessary together to coordinate and facilitate capillary assembly and maturation. Overall, this work is critical for our basic understanding of capillary formation, but also for the ability to reproducibly generate stabilized networks of capillary tubes.

## Introduction

Despite their importance, capillaries are an understudied blood vessel type. They are thinly fragile, very numerous and difficult to extract from tissues and evaluate side-by-side with larger blood vessels. Capillary tube networks consist of endothelial cell-lined tubes that are invested with pericytes found on their abluminal surface (Adams and Alitalo, 2007; Bowers et al., 2016; Kemp et al., 2022; Davis and Kemp, 2023; Lin and Davis, 2023; Spurgin and Cleaver, 2024; Yrigoin and Davis, 2024). Together, ECs and pericytes co-contribute to the deposition of the capillary basement membrane matrix, an important extracellular matrix (ECM) structure necessary for capillary maturation and stabilization (Stratman et al., 2009a; Stratman and Davis, 2012; Davis and Kemp, 2023). Capillaries are critical vessels for oxygen and nutrient exchange for all vascularized tissues, but they also contribute angiocrine signals to facilitate tissue development, maturation, specialization and stabilization (Ramasamy et al., 2015; Rafii et al., 2016; Augustin and Koh, 2017). We have proposed that healthy capillaries are disease suppressors in that they can deliver signals to surrounding cell types to inhibit disease initiation by affecting processes such as inflammation, vascular permeability, hemorrhage, thrombosis, fibrosis and autoimmunity (Davis et al., 2015; Davis and Kemp, 2023).

Considerable progress has occurred in our understanding of the biology of EC morphogenesis including lumen and tube formation, EC sprouting behavior, and pericyte recruitment to EC tubes to induce basement membrane matrix assembly (Stratman and Davis, 2012; Duran et al., 2017; Lin et al., 2021; Davis and Kemp, 2023). Critical growth factor requirements for both ECs and pericytes to co-assemble have been identified and many key signaling pathways between them are known to control EC lumen formation, sprouting behavior, and pericyte recruitment (Stratman et al., 2011; Bowers et al., 2016; Bowers et al., 2020; Kemp et al., 2020; Kemp et al., 2022). Despite these advances, it remains challenging to create networks of human EC tube networks with associated pericytes that are stable over time in 3D collagen or fibrin matrices (Park and Gerecht, 2014; Blatchley and Gerecht, 2020; Ryan and Cleaver, 2022). In our experience, there is a clear tendency for EC morphogenesis to continue in these model systems and therefore the tubes become too wide and eventually decline over time even when pericytes are present. Thus, there is an important need to further advance our knowledge of capillary assembly, such that more stable capillary tubes can be reproducibly generated. A problem in bioengineered tissue constructs include the degeneration of the associated vasculature (Hanjaya-Putra et al., 2013; Ryan et al., 2021), and overall, the factors or signals that promote long-term capillary network stability are not well understood. In previous studies, we and others identified an important role for matrix metalloproteinase (MMP) inhibitors, the tissue of metalloproteinases (TIMP)-2 and TIMP-3, in negatively controlling EC tube morphogenesis (Bayless and Davis, 2003; Seo et al., 2003; Stetler-Stevenson and Seo, 2005; Stratman et al., 2009b) and in playing a role in EC-pericyte interactions that affect both capillary assembly and stabilization (Saunders et al., 2006). In this past work, these findings suggested that EC-derived TIMP-2 and pericyte-derived TIMP-3 were important for capillary stabilization (Seo et al., 2003; Stetler-Stevenson and Seo, 2005; Saunders et al., 2006; Schrimpf et al., 2012). TIMP-3 is unique among the TIMPs in that it can interact with extracellular matrix (ECM) proteins such as perlecan due to its heparan sulfate chains (Leco et al., 1994), and it can block the activity of MMPs, but also a disintegrin and metalloproteinases (Adam) and a disintegrin and metalloproteinase with thrombospondin motifs (Adamts) proteinases (Brew et al., 2000; Murphy et al., 2003). Key metalloproteinases that are inhibited by TIMP-3 include: MMP-14, a major regulator of EC lumen formation and sprouting behavior (Hiraoka et al., 1998; Bayless and Davis, 2003; Chun et al., 2004; Saunders et al., 2006; Stratman et al., 2009b), Adam-17, a sheddase that releases key growth factors such as HB-EGF (Le Gall et al., 2010; Hewing et al., 2013), Adam-10, a required enzyme for Notch activation (Hartmann et al., 2002) and thus, a key regulator of vascular development and maturation events (Glomski et al., 2011; Alabi et al., 2016; Farber et al., 2019), and Adamts4, an enzyme that degrades the hyaluronan-binding proteoglycans, versican and aggrecan (Wayne et al., 2007). Each of these TIMP-3 targets are important to the development and function of the vasculature, thus the expression of TIMP-3 in vascular cells such as pericytes or vascular smooth muscle cells is of clear significance.

In this study, we hypothesized that upregulation of pericyte TIMP-3 might enhance capillary assembly by facilitating the creation of better and more stable capillary tube networks in 3D matrices. For this work, we developed a doxycycline-regulated TIMP-3 pericyte line and demonstrated strong upregulation of TIMP-3 expression following doxycycline addition compared to controls. EC only cultures were established in the absence or presence of doxycycline and these showed no differences. Also, they demonstrated widened and shortened tubes in both instances. In contrast, doxycycline addition to the EC-pericyte co-cultures using the TIMP-3 pericytes demonstrated marked narrowing of EC tube widths, elongated tube lengths, enhanced tube branching, and enhanced basement membrane deposition compared to co-cultures without doxycycline addition. Furthermore, the doxycycline-induced co-cultures demonstrated EC tube widths averaging 8 µm in diameter compared to 23 µm without addition, while EC only culture widths averaged about 36 µm with or without doxycycline. Finally, we demonstrated that TIMP-3 induction by itself was insufficient for this response, since blockade of pericyte recruitment even in the presence of doxycycline did not lead to narrow and elongated tubes with strong basement membrane deposition. In contrast, our results suggest that pericyte recruitment is required in conjunction with pericyte TIMP-3 induction to affect, enhance, and stabilize capillary tube network assembly. Thus, we conclude that regulated induction of pericyte TIMP-3 markedly stimulates human EC-pericyte tube network co-assembly creating physiologic capillary tube widths with very prominent deposition of basement membrane matrices, a key indicator of capillary maturation and stability.

## Materials and methods

The authors declare that all supporting data are available within the article (and its Supplementary Material).

### Cell culture

The original cell lines used in this study were either obtained from Lonza (Basel, Switzerland) or ScienCell (Carlsbad, CA), where we purchased human ECs (human umbilical vein) or pericytes (human brain vascular pericytes), respectively. Specifically, human ECs were used from passage 3 to 6, while TetOne TIMP-3 and green fluorescent protein (GFP)-labelled human pericytes were used from passage 5 to 10. All cells were passaged on gelatin-coated flasks and grown in our own Super-media, with Medium 199 as a base (ThermoFisher, Waltham, MA), 20% fetal bovine serum, bovine hypothalamus extract, heparin sodium salt, gentamicin, and amphotericin B as described (Koh et al., 2008). Cells were grown in 5% CO_2_ incubators set at 37°C.

### Lentiviral vector cloning and preparation of TetOne TIMP-3 pericyte cell line

The pLVX-TetOne-Puro system was from Clontech Takara (San Jose, CA). *EcoR1* and *BamH1* restriction enzymes were from New England Biolabs (Ipswich, MA). We used a TIMP-3 pAdTrack clone to amplify the gene for TetOne cloning using the primers TIMP-3 EcoR1 UP (5′-AGG​AAT​TCA​CCA​TGA​CCC​CTT​GGC​TCG​GGC​TCA​TC-3′) and TIMP-3 BamH1 DN (5′-AGG​GAT​CCT​CAG​GGG​TCT​GTG​GCA​TTG​ATG​ATG-3′). PCR Master Mix was from New England Biolabs. Positive plasmids were selected and confirmed expression using RT-PCR and Western blotting using an anti-TIMP-3 antibody (Sigma-Aldrich, MAB3318).

Human embryonic kidney cells (HEK293T) were maintained in Dulbecco’s modified Eagle medium (DMEM) with high glucose (ThermoFisher) supplemented with 10% fetal bovine serum, 500 μg/mL G418, gentamicin, and amphotericin B, at 37 °C and 5% CO_2_. After 70%–80% confluence, positive TetOne-TIMP-3 plasmids were packaged into Lenti-X Packaging Single Shots (Clontech Takara) and transfected to HEK293T cells. After 72 h incubation, lentivirus was harvested and transduced to low passage GFP-HBVP cells with 10 μg/mL polybrene (Sigma-Aldrich, St. Louis, MO). Positive TIMP-3-GFP-HBVP were selected using puromycin selection.

### Total RNA isolation and qPCR analysis

After reaching confluency, HBVP cultures were lysed using TRIzol (Zymo Research, Irvine, CA, #R2051) to isolate total RNA. cDNA was produced utilizing the Protoscript–First strand cDNA synthesis kit, #E6300s. (New England Biolabs). qPCR was performed using TaqMan Fast Advanced Master Mix (2x) and the endogenous control gene *gapdh* TaqMan Assay (Hs02786624_g1) (ThermoFisher). Multiplex analysis was performed to compare levels of *timp3* (Hs00165949_m1) (ThermoFisher) to endogenous control. Reactions were mixed with corresponding cDNA samples, and then placed in 96-well 0.1 mL plates for analysis using an Applied Biosystems Quant Studio 3 real-time PCR machine (ThermoFisher).

### Vessel network assembly assay

Human umbilical vein endothelial cells (at 2×10^6^ cells/mL) and TetOne TIMP-3 GFP-labelled human brain vascular pericytes were cocultured in 2.5 mg/mL type I collagen matrices in 96-well half area plates (ThermoFisher). After polymerization, cocultures were fed with 1x M199 media containing RSII (+insulin), FGF (fibroblast growth factor)-2 at 50 ng/mL, SCF (stem cell factor) at 40 ng/mL, IL-(interleukin)-3 at 40 ng/mL, and SDF (stromal cell-derived factor)-1α at 40 ng/mL. FGF-2 was obtained from Gibco (Grand Island, NY), while SCF, IL-3 and SDF-1α were obtained from R&D Systems (Minneapolis, MN). On plastic dishes or in three-dimensional (3D) collagen matrices, TIMP-3 was induced by adding 1 μg/mL doxycycline (Tocris, Bristol, UK). These 3D cultures were incubated at 37°C and assembled for a period of 0–72 h or 0–120 h and then fixed in 3% paraformaldehyde. To quantify mural cell recruitment, 5 pictures were taken from predetermined locations per experiment fixed at 72 h, and ≥3 validating experimental replicates were conducted in total for all quantitative data. Pericyte recruitment was assessed in the following way: (1) To be recruited, most of the mural cell body must be on the EC tube and (2) the mural cell must be elongated on that tube (rounded up cells did not qualify as recruited). To quantify vessel width and length 120 h co-cultures were fixed with 3% glutaraldehyde and stained with 0.1% toluidine blue in 30% methanol for nonfluorescent imaging. Four images per well and ≥3 wells per condition were taken. Widths and lengths were measured and analyzed using MetaMorph software, version 7.8 (Molecular Devices, San Jose, CA).

### Immunostaining of 3D cultures

Collagen gels were fixed in 3% paraformaldehyde, and then washed in a Tris-glycine buffer solution for at least 1 h. In some cases, a 1% Triton-X100 solution was added for 1 h. To analyze basement membrane deposition, we did not permeabilize the fixed cultures. Gels were then put into a blocking solution (Tris-buffered saline (TBS) containing 1% bovine serum albumin) and containing 5% serum specific to the secondary antibody (either goat or rabbit serum) for 1–2 h. The primary antibody was then added directly into the blocking solution and allowed to incubate overnight. After this incubation, gels were washed 3–4 times with TBS. The blocking solution containing 5% serum was added with a fluorescent secondary antibody. After 2 h, this solution was removed and washed again 3–4 times over several hours with TBS. Samples could then be imaged by immunofluorescence microscopy. For immunostaining of basement membrane matrix components, we utilized the following antibodies: Fibronectin, 1:200 dilution (Rockland, Philadelphia, PA, #6004011170.5), Laminin, 1:200 dilution (Sigma, St. Louis, MO, #071M4867), Perlecan, 1:200 dilution (Invitrogen, Carlsbad, CA, #134400), Collagen IV, derived from M3F7 hybridoma cells, 1:4 dilution (Developmental Studies Hybridoma Bank, Iowa City, IA), Nidogen-1, 1:200 dilution (R&D Systems, #AF2570), Nidogen-2 1:200 dilution (R&D Systems, #AF3385). To image ECs, we immunostained cultures with anti-CD31 antibodies, 1:200 dilution (Dako, Glostrup, Norway, #M0823). In this latter case, we also added 1% Triton X-100 (Sigma) to the blocking buffer solutions.

### Microscopy and imaging

Images for quantification of pericyte recruitment and pericyte invasion were obtained using transmitted light images of ECs, which were then overlaid with fluorescent GFP-labeled pericytes using an Olympus CKX41 microscope (Olympus, Center Valley, PA) with imaging software (DP Controller/DP Manager version 3.2.1.276). All images were obtained using a ×10 HC PL APO, 0.4NA, WD 2.2 mm lens. Confocal reconstructions and imaging were created using either LAS X (Leica Microsystems, Deerfield, IL) or Fiji (ImageJ, version 1.45f). Confocal images were obtained using a Leica SP8 LIGHTNING White light laser confocal scanning microscope. Confocal microscope images shown are representative of at least four images that are taken of each gel with each antibody used.

Time-lapse movies of 3D cultures were obtained using a DMI6000B microscope with environmental chamber (Leica Microsystems) and controlled using LAS X 3.8.26810.1 software. A ×10 objective was used for all movies. To create time-lapse movies, images were taken every 15 min with a Leica K8 CMOS camera (Leica Microsystems) in different stage locations over a period of 72–120 h. These images could then be compiled into a movie using LAS X software.

## Statistics

Student’s *t* tests were performed using Prism 10 (GraphPad Software, Boston, MA) to assess statistical significance between means in the various experiments and different conditions. When we needed to compare the means of multiple conditions within a given experiment, GraphPad (Prism 10) utilized ANOVA with follow up *post hoc* Tukey tests. The alpha value = 0.05 was set as the minimum level of statistical significance. All experiments were performed with ≥3 experimental replicates in total and each culture condition was performed with at least triplicate wells for every experiment.

## Results

### Generation of a human pericyte cell line carrying a doxycycline-inducible metalloproteinase inhibitor, TIMP-3

We previously reported that we were able to utilize human pericytes and engineer them to induce the expression of transgenes such as mCherry using a doxycycline-induced vector system during EC-pericyte tube co-assembly (Bowers et al., 2015). In the present work, we have used a similar but different lentiviral vector, pLVX-TetOne, to clone and express TIMP-3, such that we can induce its expression in pericytes upon doxycycline addition. In this manner, we could assess whether inducing the expression of TIMP-3 would have an impact on capillary morphogenesis during EC-pericyte tube co-assembly. Previously, we reported that siRNA suppression of TIMP-3 in pericytes resulted in abnormally wide and destabilized tubes that were more susceptible to regression (Saunders et al., 2006; Stratman et al., 2009a). In this work, we have done the reverse experiment and increased TIMP-3 expression in pericytes during capillary tube assembly of ECs and pericytes in 3D collagen matrices (see below).

**FIGURE 1:**
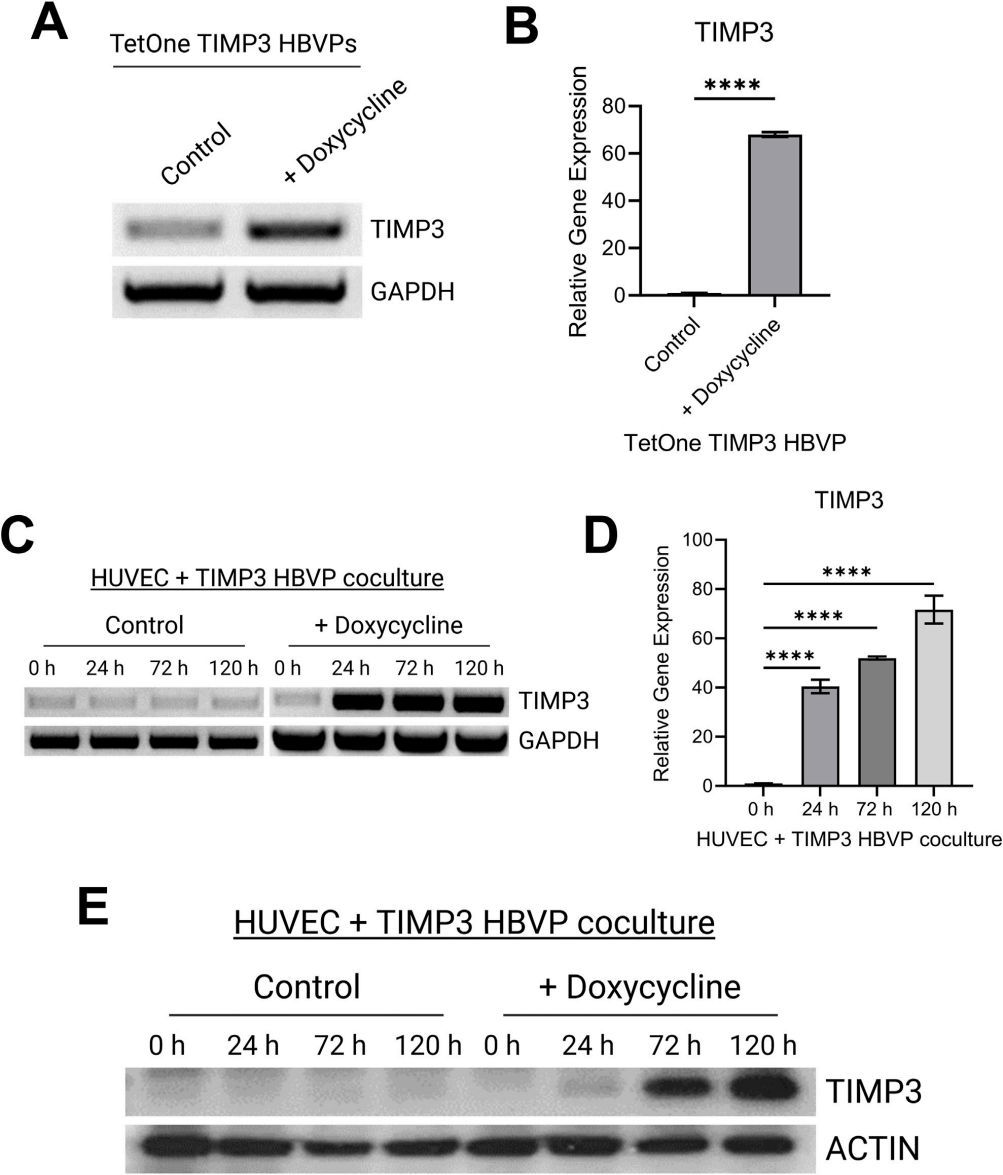
Development of a pericyte cell line carrying a doxycycline-inducible TIMP-3 construct that allows for induction of pericyte-derived TIMP-3 during capillary assembly. **(A)** RT-PCR analysis of TIMP-3 mRNA expression in pericytes with or without doxycycline treatment over 48 h. GAPDH expression is shown as a control. **(B)** Quantitative PCR analysis of TIMP-3 induction in pericytes carrying a TIMP-3 transgene in the absence or presence of doxycycline addition. GAPDH was used as an endogenous control. (n = 3); **** indicates significance at *p* ≤ 0.0001. **(C)** RT-PCR analysis of TIMP-3 mRNA expression in EC-pericyte 3D co-cultures in the absence or presence of doxycycline over the indicated time course. GAPDH expression was evaluated as a control. **(D)** Quantitative PCR analysis of TIMP-3 induction in EC-pericyte 3D co-cultures in the absence or presence of doxycycline addition over the indicated times in culture. GAPDH was used as an endogenous control. (n = 3); **** indicates significance at *p* ≤ 0.0001. **(E)** EC-pericyte co-cultures were established in 3D collagen matrices and in the absence or presence of doxycycline. At the indicated time points, lysates were prepared, and Western blots were performed to detect TIMP-3 or actin controls.

Addition of doxycycline to the engineered pericytes in 2D culture led to strong upregulation of TIMP-3 mRNA, compared to control, as detected by either RT-PCR or qPCR (Figures 1A, B). We performed the same experiment using pericytes that were co-cultured with ECs in 3D collagen matrices over a 120 h period (Figures 1C, D). A key point is that ECs do not express TIMP-3 before or during capillary assembly (Saunders et al., 2006). Addition of doxycycline leads to marked upregulation of pericyte TIMP-3 in the 3D co-cultures as detected using RT-PCR as well as qPCR analysis (Figures 1C, D). Finally, we performed Western blot analysis to demonstrate strong induction of TIMP-3 protein, following doxycycline treatment over time, compared to actin controls (Figure 1E). The TIMP-3 induction becomes particularly apparent after 72 and 120 h of culture.

### Doxycycline-induced expression of pericyte TIMP-3 enhances capillary assembly and maturation events

**FIGURE 2:**
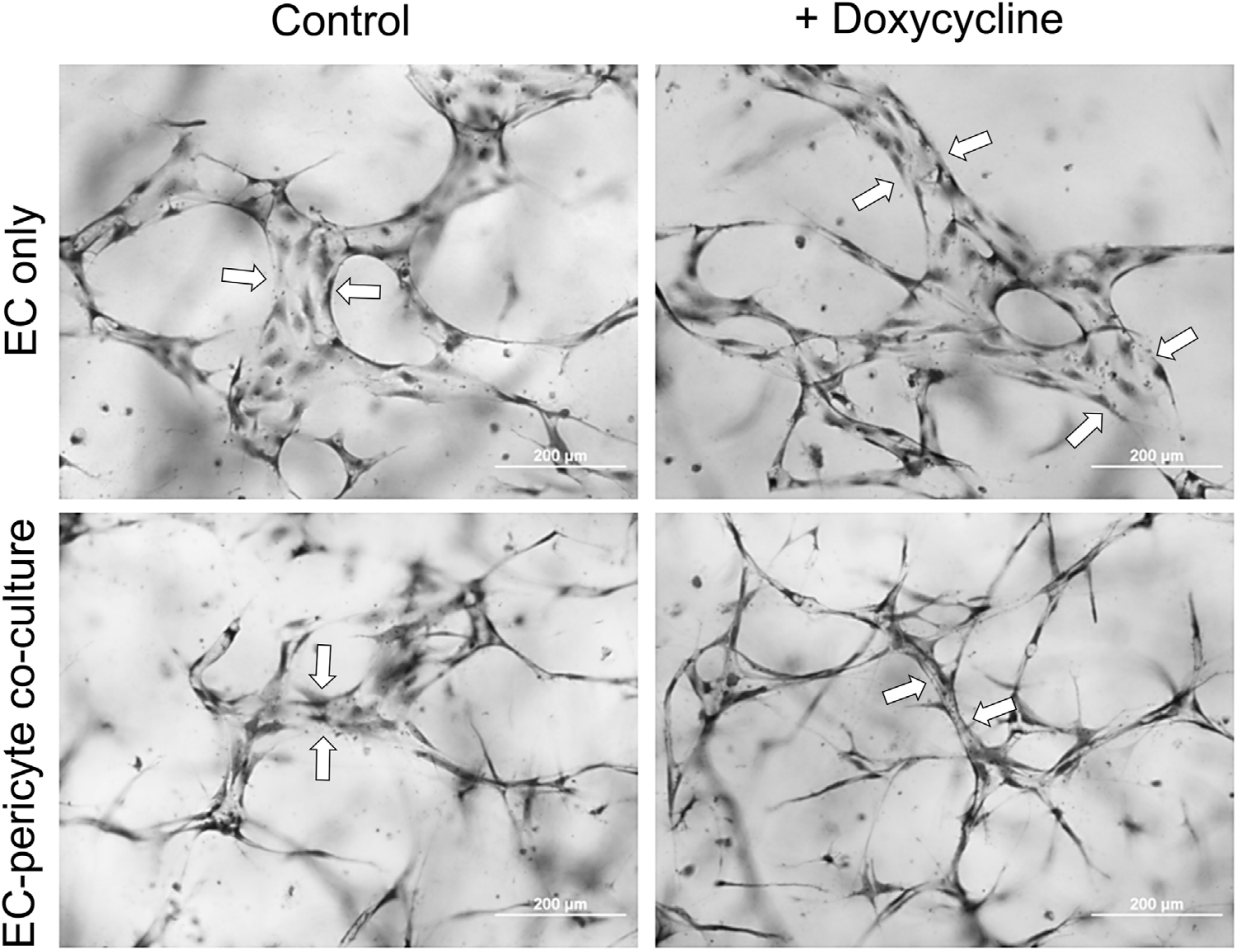
Doxycycline treatment results in marked capillary tube narrowing and elongation selectively in EC-pericyte co-cultures where the pericytes carry a doxycycline-inducible TIMP-3 transgene. EC only or EC-pericyte co-cultures were established in 3D collagen matrices, and the culture media either contained doxycycline or not. After 120 h, cultures were fixed, stained with toluidine blue and photographed. Bar equals 200 µm. Arrows indicate the borders of EC tubes revealing the varying tube widths among the different conditions.

**FIGURE 3:**
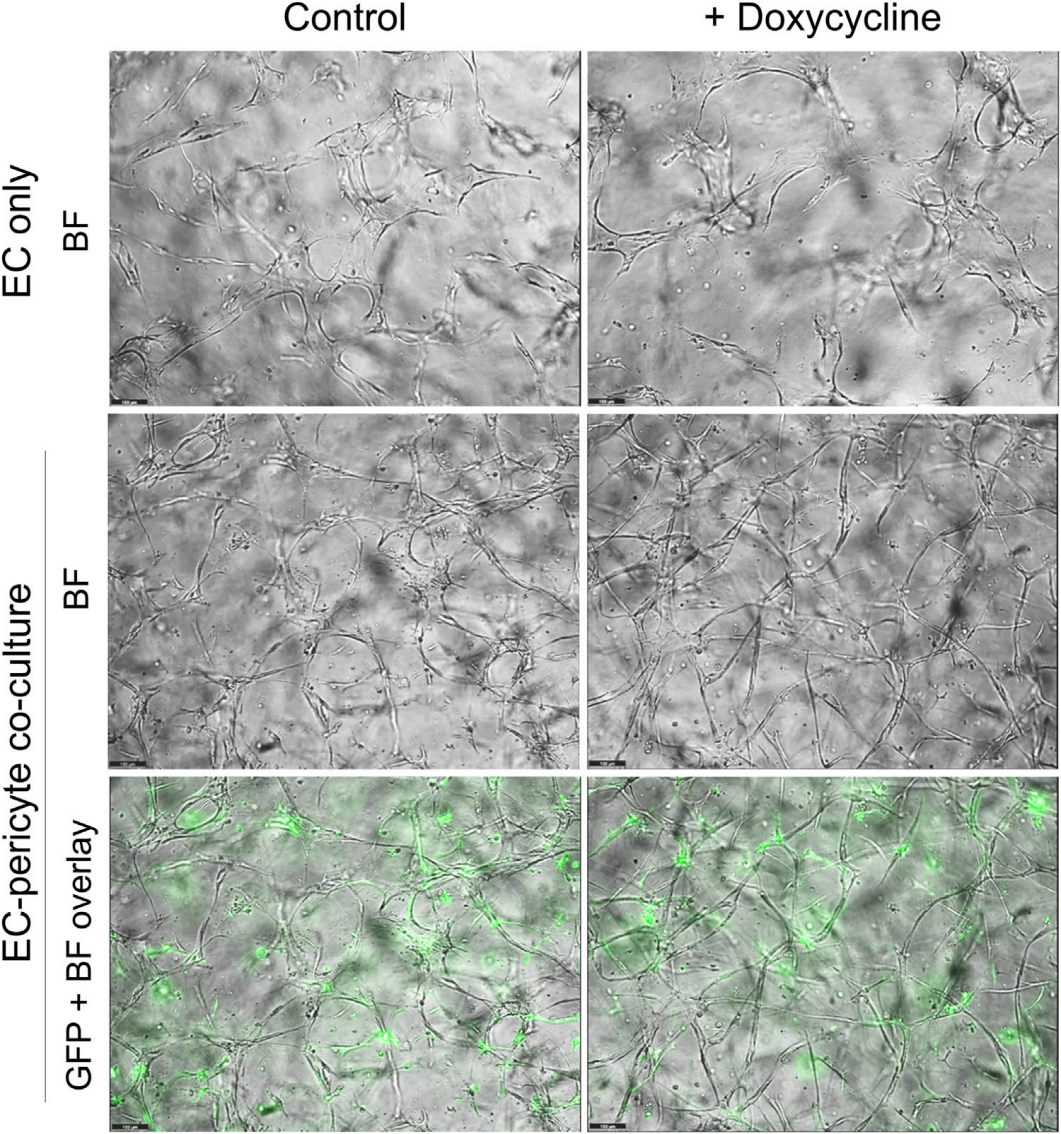
Doxycycline treatment results in marked and selective capillary tube narrowing and elongation from EC-pericyte co-cultures where the pericytes carry GFP as well as a doxycycline-inducible TIMP-3 transgene. EC only or EC-pericyte co-cultures were established in 3D collagen matrices, and the culture media either contained doxycycline or not. The pericytes carry both GFP and a doxycycline-inducible TIMP-3 transgene. Real time videos were performed and at 120 h, representative images are shown that were obtained by brightfield (BF) or fluorescence microscopy to detect GFP-labeled pericytes. The last row shows overlay images of the BF and fluorescent GFP images. Bar equals 100 µm.

**FIGURE 4:**
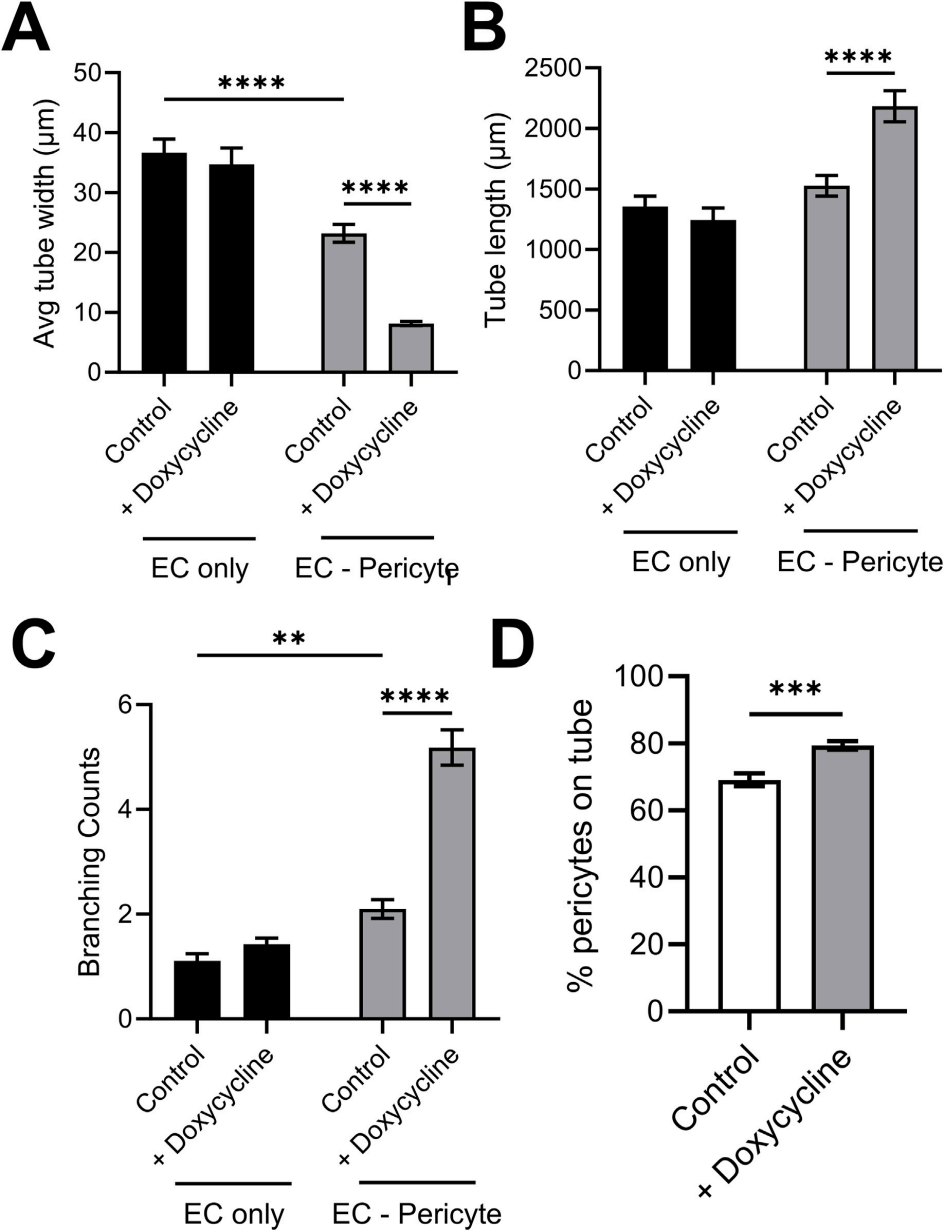
Doxycycline-induced upregulation of pericyte TIMP-3 in EC-pericyte co-cultures leads to marked EC tube narrowing, increased tube lengths, increased tube branching and increased pericyte recruitment compared to controls in the absence of doxycycline. EC only and EC-pericyte co-cultures were established in 3D collagen matrices in the absence or presence of doxycycline. The pericytes contained a TIMP-3 transgene which is inducible following doxycycline addition. After 120 h, cultures were fixed and analyzed for EC tube diameter **(A)**, EC tube lengths **(B)**, EC tube branching **(C)**, and the percentage of pericytes that have recruited to EC tubes **(D)**. ** indicates significance at *p* ≤ 0.01; *** indicates significance at *p* ≤ 0.001; **** indicates significance at *p* ≤ 0.0001.

Using this new approach to upregulate pericyte TIMP-3 expression, we performed EC only vs EC-pericyte morphogenesis assays in 3D collagen matrices to evaluate the impact of doxycycline addition as well as pericyte TIMP-3 induction on these processes (Figure 2). Still images from real-time videos of these four different experimental conditions are shown to further illustrate these results (Figure 3). EC only cultures demonstrate networks of wider tubes with a diameter of about 36 μm, without or with doxycycline addition (Figures 2, 3; Table 1). By contrast, EC-pericyte co-cultures display much thinner and more elongated tubes demonstrating diameters of 23 µm without doxycycline addition and 8 µm with doxycycline addition (Figures 2, 3, 4A; Table 1). A key point is that doxycycline treatment of these co-cultures dramatically leads to narrow capillary widths that are physiologic in diameter as capillaries *in vivo* typically 5–10 µm in diameter (Table 1). Thus, there was no impact of doxycycline addition on EC only tube formation or widths, while addition of doxycycline to EC-pericyte co-cultures using the pericytes carrying the TIMP-3 transgene caused marked narrowing of the EC tube widths (Figures 2, 3; Supplementary Figure S1; Table 1). Furthermore, addition of doxycycline to EC-pericyte co-cultures in the absence of the TIMP-3 transgene had no effect on EC tube widths or lengths (Supplementary Figure S2). Also, we examined the cultures for effects on EC tube lengths, tube branching points, and pericyte recruitment percentage (Figures 4B–D). In the EC only cultures, addition of doxycycline had no effect on either tube lengths or tube branching points (Figures 4B, C). By contrast, the EC-pericyte co-cultures demonstrated significant increases in EC tube lengths, tube branching points, and pericyte recruitment to EC tubes following treatment with doxycycline compared to controls (Figures 4B–D).

**TABLE 1:** The presence of pericytes and induction of TIMP-3 expression in pericytes markedly narrows EC tube widths during capillary assembly.

Coculture	Condition	Average tube width (μm)	± SEM (μm)
EC only	Control	36.6	2.2
	+ Doxycycline	35.5	2.6
EC-pericyte	Control	23.1	1.4
	+ Doxycycline	8.1	0.3

EC, only and EC-pericyte co-cultures were established in the presence of Factor media and in the absence or presence of doxycycline at 1 μg/mL. Cultures were fixed after 120 h, were stained with toluidine blue and EC, tube widths were quantitated.

### Real-time video visualization of doxycycline treatment of EC only and EC-pericyte co-cultures carrying the TIMP-3 transgene

To further evaluate these culture conditions, we performed real-time videos to visualize the biological effects of doxycycline addition and the induction of pericyte TIMP-3. The cultures were examined from 72–120 h and revealed that EC only cultures demonstrated branching but widened tubes that were the same in the presence or absence of doxycycline addition (Supplemental Videos S1–4). In contrast, EC-pericyte co-cultures showed branched tube networks that were narrower than EC only tubes in the absence of doxycycline (Supplemental Videos S5, 6), but became dramatically more narrowed, elongated, and branched in the presence of doxycycline (Supplemental Videos S7, 8) due to the induction of TIMP-3 expression. We also show videos demonstrating light images overlaid with green-fluorescent images to visualize GFP-pericyte behavior over this time frame. Pericytes are observed interacting with tubes in a similar manner in the absence or presence of doxycycline (Supplemental Videos S9, 10). Still brightfield and brightfield and fluorescent overlay images from these cultures at 120 h illustrate the marked differences observed under these varying conditions (Figure 3). In addition, we also examined the different cultures and conditions above using real-time videos at earlier time points. In these videos, the same conclusions are reached in that addition of doxycycline induces tube narrowing and elongation selectively in EC-pericyte co-cultures carrying the TIMP-3 transgene but not from the EC only cultures (Supplemental Videos S11, 12). Furthermore, we also show videos demonstrating light images overlaid with green-fluorescent images to visualize GFP-pericyte behavior over this earlier time frame (Supplemental Videos S13, 14).

### Enhancement of capillary basement membrane matrix deposition following doxycycline induction of pericyte-derived TIMP-3

**FIGURE 5:**
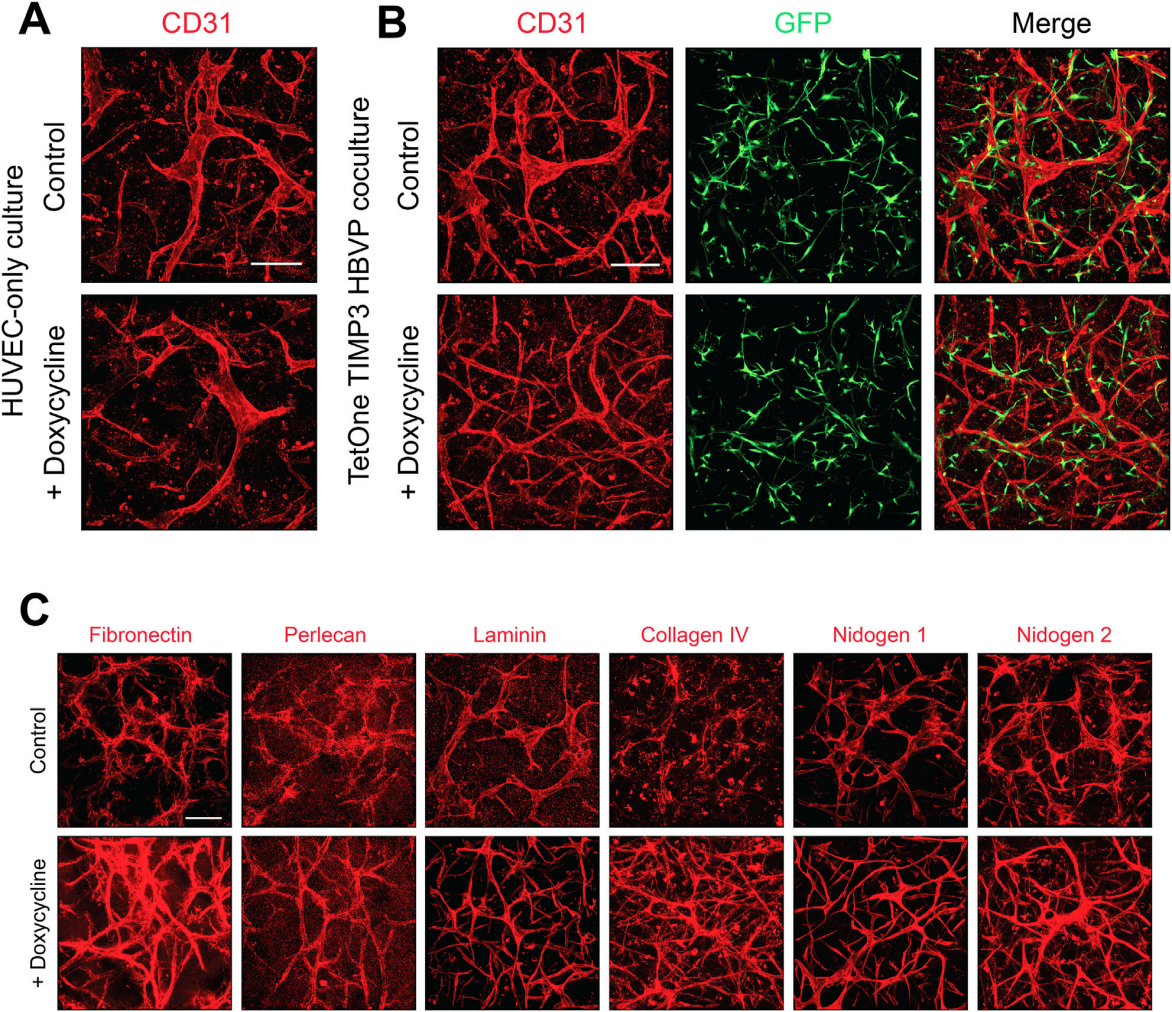
Doxycycline-induced upregulation of pericyte TIMP-3 in EC-pericyte co-cultures leads to enhanced basement membrane deposition compared to controls without doxycycline addition. **(A)** EC only cultures were seeded in 3D collagen matrices in the absence or presence of doxycycline. At 120 h, cultures were fixed, immunostained with anti-CD31 antibodies, and imaged by confocal microscopy. **(B)** EC-pericyte co-cultures were seeded in 3D collagen matrices in the absence or presence of doxycycline and after 120 h were fixed and immunostained with anti-CD31 antibodies and, imaged by confocal microscopy. The pericytes contained a TIMP-3 transgene which is inducible following doxycycline addition. **(C)** EC-pericyte co-cultures were seeded in 3D collagen matrices in the absence or presence of doxycycline and after 120 h were fixed and immunostained with the indicated basement membrane matrix antibodies and, imaged by confocal microscopy. In each case, representative images of our results are shown. Bar equals 200 µm.

We have reported that a major consequence of EC-pericyte interactions during capillary formation results in deposition of the basement membrane matrix (Stratman et al., 2009a). EC only cultures do not deposit basement membrane matrices in the absence of pericytes under our defined culture conditions. We initially immunostained cultures of EC only (Figure 5A) and EC-pericyte co-cultures (Figure 5B) treated in the absence or presence doxycycline. Staining with anti-CD31 antibodies reveals widened and shortened EC tubes in the EC only cultures with or without doxycycline addition, while EC-pericyte co-cultures are markedly thinner and more elongated which was particularly evident following doxycycline addition.

As expected, pericyte recruitment to developing tubes regulates the deposition of basement membrane matrix components in the control EC-pericyte co-cultures in the absence of doxycycline addition (Figure 5C). However, doxycycline addition strongly induces the deposition of collagen type IV, fibronectin and nidogen 2 in these EC-pericyte co-cultures utilizing pericytes carrying the TIMP-3 transgene (Figure 5C). Previously, we reported that fibronectin assembly may be functionally involved in collagen type IV deposition (Stratman et al., 2009a), and possibly, vice-versa. Furthermore, other studies have also implicated a role for fibronectin in deposition of collagen type I fibrils (Kubow et al., 2015).

### Blockade of pericyte recruitment interferes with the increased basement membrane matrix deposition in response to doxycycline treatment in EC-pericyte co-cultures

**FIGURE 6:**
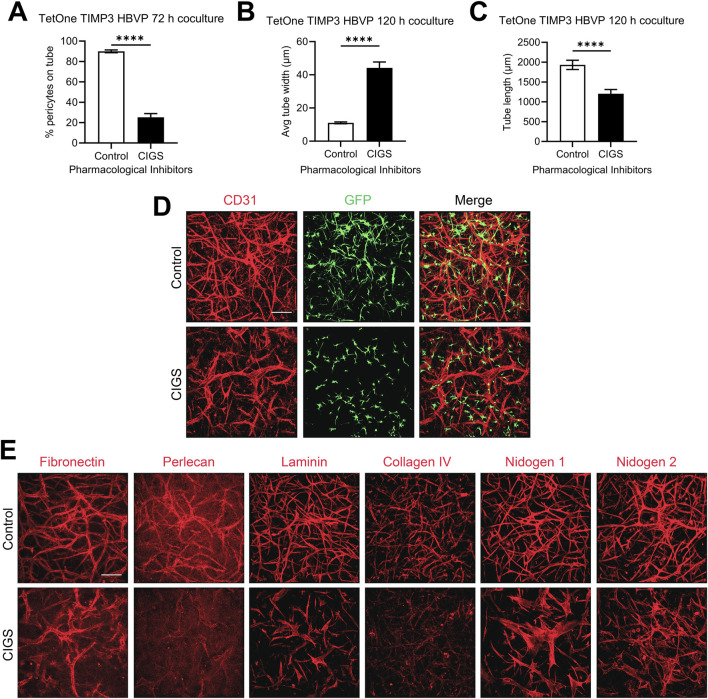
Blockade of pericyte recruitment using pharmacological inhibitors interferes with the impact of doxycycline induction of pericyte TIMP-3 resulting in disrupted capillary assembly with diminished pericyte recruitment, widened EC tube widths, decreased tube lengths, and strongly reduced basement membrane matrix deposition. EC-pericyte co-cultures were established using 3D collagen matrices in the absence or presence of the pharmacological drug combination, CIGS, which is known to strongly reduce pericyte recruitment. CIGS = CI 1020 (20 µM); Imatinib (1 µM); Gefitinib (1 µM); SB431542 (10 µM). Cultures were fixed after 120 h and were quantitated for the percentage of pericyte recruitment to tubes **(A)**, EC tube widths **(B)**, and EC tube lengths **(C)**. In addition, immunostaining was performed with anti-CD31 antibodies to label ECs **(D)**, and with antibodies to the indicated basement membrane matrix components **(E)**. In each case, representative images of our results are shown. Bar equals 200 µm.

To examine these results further, we utilized pharmacological agents that we previously reported could block pericyte recruitment during capillary EC tube network assembly. This drug combination, abbreviated CIGS, utilizes four drugs, which inhibit the pericyte receptors for EC-derived factors that are necessary for pericyte recruitment including PDGF-BB, PDGF-DD, endothelin-1, TGFβ1, and HB-EGF (Kemp et al., 2020). CIGS addition blocks pericyte responsiveness to these factors and they fail to elongate and invade in 3D collagen matrices (Kemp et al., 2020). Using the pericytes carrying the TIMP-3 transgene and in the presence of doxycycline, we evaluated co-cultures in the absence or presence of added CIGS and demonstrate that CIGS addition markedly interferes with pericyte recruitment (Figure 6A), which then leads to increases in EC tube widths (Figure 6B) and decreases in EC tube lengths (Figure 6C). The tube network morphogenic differences are demonstrated by anti-CD31 immunostaining of control vs CIGS-treated co-cultures (Figure 6D). Next, we evaluated basement membrane matrix deposition, which demonstrated diminished deposition of the different components. This decrease in basement membrane components was particularly evident with perlecan, laminin and collagen type IV (Figure 6E). These findings suggest that induction of pericyte TIMP-3 and the recruitment of pericytes to EC-lined tubes are coupled together to co-regulate the marked morphologic and basement membrane matrix deposition events that are observed. Furthermore, this result suggests that induction of pericyte TIMP-3 is insufficient by itself to induce these dramatic changes and requires concomitant pericyte recruitment and interactions with EC-lined tubes. Also, enhanced deposition of the capillary basement membrane matrix likely plays a role in restricting further luminal expansion, which contributes to the maintenance of an elongated and narrow capillary tube structure. The known ability of TIMP-3 to interact with basement membrane components like perlecan might concentrate TIMP-3 in the basement membrane to facilitate its ability to block invasive behavior from either ECs or pericytes, to restrict further morphogenesis and promote capillary stabilization.

To further support this conclusion, we performed a second experiment to interfere with pericyte recruitment using a combination of neutralizing antibodies directed to PDGF-BB, PDGF-DD, and HB-EGF, a receptor trap for TGFβ1 (i.e., recombinant Alk5-Fc), and a chemical inhibitor of endothelin receptor A (termed neutralizing agents) (Supplemental Figure S3). Using these neutralizing agents, pericyte recruitment was partially inhibited resulting in wider EC tube widths and shorter EC tube lengths compared to controls (Supplemental Figure S3A–D). In addition, basement membrane deposition was reduced comparing the neutralizing agent co-cultures with the controls (Supplemental Figure 3E) in a manner very similar to the CIGS addition experiment (Figure 6). Very striking diminishment of fibronectin, collagen type IV, perlecan and nidogen 2 were observed when pericyte recruitment was inhibited by the neutralizing agents (Supplemental Figure 3E). This additional data provides more support for the concept that induction of pericyte TIMP-3 must be directly coupled to pericyte recruitment to strongly facilitate stable capillary network assembly.

## Discussion

In recent years, considerable progress has been made in elucidating the basic biology underlying how ECs form lumens and tubes, and then interact with pericytes to induce stabilization of capillary tube networks (Stratman et al., 2009a; Bowers et al., 2016; Bowers et al., 2020; Kemp et al., 2020; Kemp et al., 2022; Ryan and Cleaver, 2022; Davis and Kemp, 2023; Spurgin and Cleaver, 2024). These EC-pericyte interactions lead to deposition of the capillary basement membrane matrix, a key step in vessel maturation and stabilization (Stratman et al., 2009a; Kemp et al., 2020). Furthermore, ECs secrete important growth factors and peptides that are necessary for the pericyte recruitment process including PDGF-BB, PDGF-DD, endothelin-1 (ET-1), TGFβ1, and HB-EGF (Kemp et al., 2020). Blockade of the pericyte receptors for these EC-derived factors or the factors themselves using neutralizing antibodies or receptor traps leads to marked impairment of pericyte recruitment, widening and shortening of EC tubes, and strongly reduced basement membrane matrix deposition (Kemp et al., 2020). Another regulator of the EC-pericyte co-assembly process are the MMP inhibitors, TIMP-2 and TIMP-3, which have been shown to play a role in capillary tube stability (Saunders et al., 2006). Both of these TIMPs have been reported to interfere with EC tube morphogenesis by blocking either lumen formation or sprouting behavior (Bayless and Davis, 2003; Saunders et al., 2006; Stratman et al., 2009b). They can also inhibit soluble MMPs such as MMP-1 and MMP-10 which have been reported to induce capillary regression by stimulating proteolysis of the ECM in which EC tubes are embedded (Saunders et al., 2005). siRNA suppression of pericyte TIMP-3 previously was reported to cause EC tube widening in EC-pericyte co-cultures and loss of collagen type IV immunostaining suggesting that basement membrane degradation occurred as a result (Stratman et al., 2009a). TIMP-3 is an ECM-binding inhibitor of MMPs, Adam, and Adamts metalloproteinases (Brew et al., 2000; Mohammed et al., 2003; Murphy et al., 2003), which is normally expressed by pericytes (Lafleur et al., 2001; Saunders et al., 2006; Stratman et al., 2009a), with much less expression by ECs. The factors or signals that regulate the expression of pericyte TIMP-3 are not well understood in a developmental or vascular maintenance context. In one study, reducing the expression of pericyte Alk-5, a TGFβ receptor, lead to reduced TIMP-3 expression and increased brain hemorrhages (Dave et al., 2018). Interestingly, pericyte TIMP-3 is known to be reduced in diabetic nephropathy and retinopathy and this appears to contribute to capillary loss and renal fibrosis in diabetes (Schrimpf et al., 2012; Wang et al., 2020). In a number of studies TIMP-3 has been administered as a therapeutic agent and has shown promise to reduce oxygen-induced retinopathy (Hewing et al., 2013) and reduce inflammatory changes in diabetic retinopathy (Abu El-Asrar et al., 2021).

In this study, we wondered if increasing the expression of TIMP-3 in pericytes might enhance capillary assembly and stabilization. To this end, we developed a human pericyte cell line carrying a TIMP-3 transgene which could be upregulated with doxycycline treatment. We first established that TIMP-3 is strongly induced following doxycycline treatment in either 2D or 3D culture. We performed 3D EC only cultures or EC-pericyte co-cultures in the absence or presence of doxycycline treatment. Doxycycline addition had no impact on EC tube formation (or tube widths) in EC only cultures compared to controls without doxycycline. In contrast, marked narrowing of EC tube widths, increased tube lengths, increased tube branching and enhanced basement membrane matrix deposition occurs when doxycycline is added to EC-pericyte co-cultures using the inducible TIMP-3 transgene carrying pericytes, compared to controls without added doxycycline. In these co-cultures, capillary widths reached an average of 8 µm with doxycycline addition (Table 1), which are in the physiological range of most capillary widths *in vivo*, which typically range from 5–10 µm. Furthermore, in order for the enhanced pericyte TIMP-3 expression to have a major impact on capillary assembly, pericyte recruitment to the EC tubes was necessary for these effects. Blockade of pericyte recruitment in this instance, caused EC tubes to widen, EC tube lengths to shorten, and basement membrane matrix deposition to be strongly reduced even when doxycycline was added. In addition, one possible explanation for our findings is that EC cell surface proteinases such as MT1-MMP and MT2-MMP, which regulate EC lumen and tube formation by degrading the collagen type I matrix (Saunders et al., 2006; Stratman et al., 2009b), also proteolytically degrade assembling type IV collagen matrices (and possibly the fibronectin matrices) during capillary assembly. This data is consistent with the idea that pericyte-induction of TIMP-3 will block these MT-MMPs, thus, interfering with EC lumen and tube expansion, but also act to enhance basement membrane matrix assembly by preventing degradation of the newly deposited collagen type IV and fibronectin matrices. This combination of effects may also lead to enhanced EC tube elongation, which may be a reason why we observed greater tube lengths and tube branching with doxycycline addition. Another possibility is that elevations in pericyte TIMP-3 could result in a greater inhibitory effect on EC lumen and tube expansion in comparison to EC sprouting behavior, which again could result in narrower tubes along with increased tube lengths and branches. Further studies will be necessary to investigate these questions.

One interpretation of these findings is that basement membrane deposition is important around capillaries to provide an inhibitory signal, which may also serve as a maturation signal that opposes further morphogenesis and, promotes tube stabilization (Davis and Senger, 2005; Davis and Kemp, 2023). TIMP-3 is known to bind heparan sulfate chains and may be decorating the basement membrane to prevent EC lumen formation and sprouting behavior, through its ability to inhibit MT (membrane type)-MMPs such as MMP-14 and MMP-15; thus, diminishing any further EC morphogenesis. Other capillary basement membrane components may also play a role in interfering with morphogenesis such as the different laminin isoforms (Davis and Senger, 2005), although their specific roles are not well understood.

Our novel findings that we can readily obtain extensive networks of human capillary tubes of physiological diameters with associated basement membranes are of great interest and represents an advance in the bioengineering of the smallest and most delicate vascular bed (i.e., capillaries) in 3D matrices. Our model system is performed under serum-free defined conditions with the defined growth factors, SCF, IL-3, SDF-1α, FGF-2 and insulin, and the system works consistently and reproducibly (Stratman et al., 2011; Bowers et al., 2016). We hope that this new model system might become useful in tissue engineering applications using different 3D matrices including either fibrin or collagen matrices, which are important matrices in postnatal angiogenic responses. There have been problems reported with the development of microvascular beds within tissue engineered constructs or organoids particularly due to vessel loss which can be observed over time (Hanjaya-Putra et al., 2013; Ryan et al., 2021). The advances that we describe in this study concerning the enhancement of capillary assembly as well as the increased deposition of basement membrane matrices might be key reasons why this new approach may improve the success and stability of such engineered tissues. Future studies should address this possibility.

Another potential application of this new technology would be to attempt to repair vascular malformations (Wetzel-Strong et al., 2017; Queisser et al., 2021; Snellings et al., 2021). Interestingly, recent studies have revealed that poor pericyte recruitment and basement membrane deposition around abnormal EC tube structures underlies the pathogenic development of vascular malformations, which represent capillary deficiency states (Sun et al., 2022; Lin et al., 2024). Examples include EC expression of activating mutations for k-Ras, and Akt1, which result in marked widening and shortening of EC tubes that is caused by accentuation of the EC lumen formation process coupled to reduced sprouting behavior (Sun et al., 2022; Lin et al., 2024). In addition, strongly reduced pericyte recruitment is observed as well as markedly diminished basement membrane deposition. Reduced pericyte recruitment occurs in part due to diminished mRNA expression of the different EC-derived factors (which attract pericytes) from the ECs carrying these gain-of-function mutations (Lin et al., 2024). Addition of the TIMP-3 transgene pericytes to ECs carrying activating mutations might be expected to diminish EC tube diameter and increase EC tube elongation if pericyte recruitment could be enhanced in some manner to the EC tubes carrying gain-of-function mutations. It is apparent that these vascular malformations are highly complex, and it will take a comprehensive understanding of the biology of capillary assembly, to have any realistic opportunity to develop an effective therapeutic approach to prevent or repair such abnormal vascular structures, but upregulation of pericyte TIMP-3 may be a possible strategy.

## Data Availability

The original contributions presented in the study are included in the article/Supplementary Material, further inquiries can be directed to the corresponding author.

## Supplementary material

SUPPLEMENTARY FIGURE S1 Doxycycline treatment results in marked capillary tube narrowing and elongation selectively in EC-pericyte co-cultures where the pericytes carry a doxycycline-inducible TIMP-3 transgene, while EC only cultures are not affected. EC only or EC-pericyte co-cultures were established in 3D collagen matrices, in the absence or presence of doxycycline. Cultures were fixed after 120 hr, and then they were stained with toluidine blue and photographed. Bar equals 200 μm. Arrows indicate the borders of EC tubes revealing the varying tube widths among the different conditions.

SUPPLEMENTARY FIGURE S2 Doxycycline treatment results in marked capillary tube narrowing and elongation selectively in EC-pericyte co-cultures where the pericytes carry a doxycycline-inducible TIMP-3 transgene, but not when control GFP-pericytes are utilized. EC-pericyte co-cultures were established in 3D collagen matrices, in the absence or presence of doxycycline. We utilized GFP-pericytes carrying a doxycycline-inducible TIMP-3 transgene or control pericytes that did not have the transgene. Cultures were fixed after 120 hr, and then they were stained with toluidine blue and photographed. EC tube widths and lengths were quantitated and are shown as a percent of the two pericyte groups in the absence of doxycycline. *** indicates significance at *p* ≤ 0.001; **** indicates significance at *p* ≤ 0.0001.

SUPPLEMENTARY FIGURE S3 Blockade of pericyte recruitment using neutralizing agents interferes with the impact of doxycycline induction of pericyte TIMP-3 resulting in disrupted capillary assembly with diminished pericyte recruitment, widened EC tube widths, decreased tube lengths, and reduced basement membrane matrix deposition. EC-pericyte co-cultures were established using 3D collagen matrices in the absence or presence of a combination of neutralizing agents. The neutralizing agents added are blocking antibodies to PDGF-BB, PDGF-DD, and HB-EGF, which were all added at 10 μg/ml. The TGFβ neutralizing agent, Alk5-Fc, was added at 10 mg/ml, and the endothelin receptor A antagonist, CI 1020 was added at 20 μM. Cultures were fixed after 120 h and were quantitated for the percentage of pericyte recruitment to tubes **(A)**, EC tube widths **(B)**, and EC tube lengths. In addition, immunostaining was performed with anti-CD31 antibodies to label ECs **(D)**, and with antibodies to the indicated basement membrane matrix components **(E)**. Bar equals 200 μm. **** indicates significance at *p* ≤ 0.0001.

SUPPLEMENTARY VIDEO S1 EC only cultures were established in the absence of doxycycline addition and imaged from 72–120 h. The video is shown at 10.7 frames per second. Bar equals 100 μm.

SUPPLEMENTARY VIDEO S2 EC only cultures were established in the absence of doxycycline addition and a second representative field was imaged from 72–120 h. The video is shown at 10.7 frames per second. Bar equals 100 μm.

SUPPLEMENTARY VIDEO S3 EC only cultures were established in the presence of doxycycline addition and imaged from 72–120 h. The video is shown at 10.7 frames per second. Bar equals 100 μm.

SUPPLEMENTARY VIDEO S4 EC only cultures were established in the presence of doxycycline addition and a second representative field was imaged from 72–120 h. The video is shown at 10.7 frames per second. Bar equals 100 μm.

SUPPLEMENTARY VIDEO S5 EC-pericyte co-cultures were established in the absence of doxycycline addition and imaged from 72–120 h. The pericytes carry a TIMP-3 transgene which is inducible following doxycycline addition. The video is shown at 10.7 frames per second. Bar equals 100 μm.

SUPPLEMENTARY VIDEO S6 EC-pericyte co-cultures were established in the absence of doxycycline addition and a second representative field was imaged from 72–120 h. The pericytes carry a TIMP-3 transgene which is inducible following doxycycline addition. The video is shown at 10.7 frames per second. Bar equals 100 μm.

SUPPLEMENTARY VIDEO S7 EC-pericyte co-cultures were established in the presence of doxycycline addition and imaged from 72–120 h. The pericytes carry a TIMP-3 transgene which is inducible following doxycycline addition. The video is shown at 10.7 frames per second. Bar equals 100 μm.

SUPPLEMENTARY VIDEO S8 EC-pericyte co-cultures were established in the presence of doxycycline addition and a second representative field was imaged from 72–120 h. The pericytes carry a TIMP-3 transgene which is inducible following doxycycline addition. The video is shown at 10.7 frames per second. Bar equals 100 μm.

SUPPLEMENTARY VIDEO S9 EC-pericyte co-cultures were established in the absence of doxycycline addition and imaged using both light and fluorescence microscopy from 72–120 h. The GFP-pericytes also carry a TIMP-3 transgene which is inducible following doxycycline addition. This video is composed of brightfield images overlaid with fluorescent GFP images. The video is shown at 10.7 frames per second. Bar equals 100 μm.

SUPPLEMENTARY VIDEO S10 EC-pericyte co-cultures were established in the presence of doxycycline addition and imaged using both light and fluorescence microscopy from 72-120 hr. The GFP-pericytes also carry a TIMP-3 transgene which is inducible following doxycycline addition. This video is composed of brightfield images overlaid with fluorescent GFP images. The video is shown at 10.7 frames per second. Bar equals 100 μm.

SUPPLEMENTARY VIDEO S11 EC only cultures were established in the absence of doxycycline addition and imaged from 0-56 hr. The video is shown at 10.2 frames per second. Bar equals 100 μm.

SUPPLEMENTARY VIDEO S12 EC only cultures were established in the presence of doxycycline addition and imaged from 0-56 hr. The video is shown at 10.2 frames per second. Bar equals 100 μm.

SUPPLEMENTARY VIDEO S13 EC-pericyte co-cultures were established in the absence of doxycycline addition and imaged using both light and fluorescence microscopy from 0-56 hr. The pericytes carry a TIMP-3 transgene which is inducible following doxycycline addition. This video is composed of brightfield images overlaid with fluorescent GFP images. The video is shown at 10.2 frames per second. Bar equals 100 μm.

SUPPLEMENTARY VIDEO S14 EC-pericyte co-cultures were established in the presence of doxycycline addition and imaged using both light and fluorescence microscopy from 0-56 hr. The pericytes carry a TIMP-3 transgene which is inducible following doxycycline addition. This video is composed of brightfield images overlaid with fluorescent GFP images. The video is shown at 10.2 frames per second. Bar equals 100 μm.
